# Supplementary material for: Alteration of the corpus callosum in patients with Alzheimer’s disease: Deep learning-based assessment
Source: PLoS One. 2021 Dec 23;16(12):e0259051. doi: 10.1371/journal.pone.0259051 (PMC8700055; doi:10.1371/journal.pone.0259051)
Supplement: S1 Table — (DOCX) [file pone.0259051.s001.docx]

Supplementary table e-1. Associations between MMSE score and each MRI measurements after controlling diagnostic group

|  | Estimate | SE | R2 | p-value |
| --- | --- | --- | --- | --- |
| **Total CC length** | -0.037 | 0.082 | 0.260 | 0.655 |
| **Genu and rostrum** |  |  |  |  |
| Width of the genu | 0.002 | 0.036 | 0.229 | 0.945 |
| Width of center between genu and rostrum | 0.001 | 0.042 | 0.168 | 0.977 |
| Width of rostrum | 0.060 | 0.076 | 0.061 | 0.427 |
| Height between the genu and rostrum | 0.001 | 0.032 | 0.069 | 0.983 |
| **Body** |  |  |  |  |
| Height of the anterior third | 0.046 | 0.012 | 0.273 | <0.0001 |
| Height of the middle third | 0.022 | 0.013 | 0.193 | 0.103 |
| Height of the posterior third | 0.015 | 0.016 | 0.143 | 0.331 |
| Width | 0.083 | 0.032 | 0.273 | 0.010 |
| **Splenium** |  |  |  |  |
| Width | 0.009 | 0.035 | 0.030 | 0.799 |
| Height | 0.031 | 0.025 | 0.381 | 0.222 |
| Area | 0.016 | 0.036 | 0.263 | 0.656 |

CC, corpus callosum; MMSE, Mini-Mental State Examination; MRI, magnetic resonance imaging; SE, standard error; R2, coefficient of determination.
