# Supplementary material for: Alteration of the corpus callosum in patients with Alzheimer’s disease: Deep learning-based assessment
Source: PLoS One. 2021 Dec 23;16(12):e0259051. doi: 10.1371/journal.pone.0259051 (PMC8700055; doi:10.1371/journal.pone.0259051)
Supplement: S1 Text — (DOCX) [file pone.0259051.s002.docx]

Supplementary text. The MATLAB-based code for extracting the corpus callosum

*a=spm_vol('c2orig0001.nii'); % real the volume*

*b=spm_read_vols(a); % read the volume into image matrix*

*data=squeeze(b(128,:,:)); % extract the sagittal slice; since data is 79x95x79, we will take 39th/40th slice*

*figure(1),imshow(data,[]); % show the image (extract_midslice.jpg)*

*bw=bwareaopen(data,100); % remove small objects; objects with pixels less than 100*

*labelbw=bwlabel(bw); % label the image*

*figure(3),imshow(bw);% display labeled image (labeled_image.jpg)*

*figure(4),imshow(labelbw==2); % extract the appropriate label image which has corpus callosum (Segmented_CC.jpg)*
