# Supplementary material for: Alteration of the corpus callosum in patients with Alzheimer’s disease: Deep learning-based assessment
Source: PLoS One. 2021 Dec 23;16(12):e0259051. doi: 10.1371/journal.pone.0259051 (PMC8700055; doi:10.1371/journal.pone.0259051)
Supplement: Attachment — Submitted filename: Reviewer_s comments_2_results.docx [file pone.0259051.s004.docx]

Review Comments to the Author

Reviewer #1: Thanks to the authors for their re-working of the manuscript. You have done a good job.

There is just one last thing that I think is necessary before acceptance. You have now presented the "training, validation, and testing" numbers. You have an accuracy of 91% with early termination.

- Firstly, it's unclear if this accuracy is reflecting the validation or testing. I assume testing, but it could be both, as it seemed to have influenced an "early stop" when, I again assume, you reached the highest accuracy level.
There's a bias in exiting training early at the highest accuracy level, as it can represent an overfitting of your own data.

Considering the above stated, in combination with the fact that you only have a small sample size (100 - which is not atypical for biomedical scenarios - and thus why you aptly chose the U-net). I would like to see a cross-validation of your data (I'd recommend at least K-fold: 10, unless you want to do leave-one-out cross-validation). This will give you a better and more true reflection of the performance of your algorithm. It is not uncommon to just do a cross-validation with training and validation data (i.e. skip the test data -> you need as much as you can for the training). Also, have a pre-set number of epochs, and choose the accuracy of the last epoch (i.e. not necessarily the "highest accuracy"). When you do the cross-validation there should not be any tuning of the hyperparameters, as this will affect the results. In the end we are interested in how your algorithm performs without having to tweak it every single time it's applied.
Also, please describe your metric "accuracy". I believe a dice-score metric would be the absolute best for your scenario, but several papers present the classic accuracy (TP + TN / TP + TN + FP + FN), as this tends to show higher accuracy.

Response:

In response to reviewer’s comment, we performed 10-fold cross-validation as follows. And we used Intersection-Over_Union (IOU) Matric for accuracy (TP/TP+FP+FN)

90% training data 🡪 divide into 10 fold, 1 fold for validation, 9 folds for training

10% test data

Used IOU(Intersection-Over_Union) Metric for “accuracy”: TP/(TP+FP+FN)

TP: true positive

FP: Flase positive

FN: False negative

Plots of accuracies and losses of validation per each k

| k | Accuracies and losses of validation data | Accuracies of test data |
| --- | --- | --- |
| 1 |  | 0.974044 |
| 2 |  | 0.974546 |
| 3 |  | 0.974053 |
| 4 |  | 0.974123 |
| 5 |  | 0.973244 |
| 6 |  | 0.974320 |
| 7 |  | 0.974123 |
| 8 |  | 0.974245 |
| 9 |  | 0.974196 |
| 10 |  | 0.973881 |
